# Message from animal models and experimental medicine for 2024—Persevering in its mission to be at the heart of laboratory animal science

**DOI:** 10.1002/ame2.12395

**Published:** 2024-03-25

**Authors:** Liu Depei

**Affiliations:** ^1^ Chinese Academy of Engineering Beijing China

In recent years, new technologies have enabled a steady stream of novel theoretical approaches to understanding the mechanisms of disease occurrence and development. The emergence of these new technologies has provided the pharmaceutical industry with a huge amount of data that can be utilized, such as brain‐computer interfaces, artificial intelligence (AI) doctors, wearable medical testing devices, etc. The continuous emergence of these new technologies and theories has facilitated the deep mining and analysis of data and the development of new medical devices. In this era of accelerated change in both old and new knowledge bases, laboratory animals are the incubator for innovative research and development of new drugs, vaccines, and medical products. Laboratory animal science has facilitated the transformation of basic medical research into clinical medicine. It is a core element of pharmaceutical research and innovation, as well as an emerging interdisciplinary field. It integrates the theories and methods of medicine, pharmacy, biology, bioengineering and other disciplines, promotes an interdisciplinary approach, and is at the forefront of research in biological science and technology. Moreover, it is the foundation of breakthroughs in original innovation and the key link to technology transformation.

*AMEM* focuses on laboratory animal science, and the journal is therefore keen to seize the opportunity to actively guide and promote the transformation of medicine. By adhering to its mission of providing scientists in the field of life sciences with a platform for international exchanges and the wide dissemination of results, and establishing itself as a highly regarded journal within the discipline, *AMEM* has become a leader in the field of laboratory animal science. To pursue its mission of innovation, the journal has created international hot topics columns including Medical Artificial Intelligence, Stem Cells and Regenerative Medicine, Neurodegenerative Diseases, Cardiovascular and Cerebrovascular Diseases and other columns with distinctive features. In 2024, *AMEM* has developed into one of the main platforms for communicating international research results.

Since its launch in March 2018, *AMEM* has been successively included in domestic and international databases such as CSCD (core), DOAJ, PubMed, WJCI, MEDLINE, ESCI, Scopus, etc. These achievements are hard‐earned and result from *AMEM*'s policy of focusing on the field of laboratory animal disease models and medical experiments and paying close attention to international developments in research, enabling *AMEM* to steadily improve its competitiveness. I am very pleased to see *AMEM* becoming a leader in the discipline and an international platform for dissemination of results.

I wish *AMEM* greater international influence in the future so that it can promote and facilitate the development of the laboratory animal science!

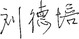

